# Decorating Nanoparticle Surface for Targeted Drug Delivery: Opportunities and Challenges

**DOI:** 10.3390/polym8030083

**Published:** 2016-03-17

**Authors:** Zhiqiang Shen, Mu-Ping Nieh, Ying Li

**Affiliations:** 1 Department of Mechanical Engineering, University of Connecticut, Storrs, CT 06269, USA; zhiqiang.shen@uconn.edu; 2 Department of Chemical and Biomolecular Engineering, Department of Biomedical Engineering and Polymer Program, Institute of Materials Science, University of Connecticut, Storrs, CT 06269, USA; mu-ping.nieh@uconn.edu; 3 Institute of Materials Science, University of Connecticut, Storrs, CT 06269, USA

**Keywords:** drug delivery, nanoparticle, surface decorating, smart polymer

## Abstract

The size, shape, stiffness (composition) and surface properties of nanoparticles (NPs) have been recognized as key design parameters for NP-mediated drug delivery platforms. Among them, the surface functionalization of NPs is of great significance for targeted drug delivery. For instance, targeting moieties are covalently coated on the surface of NPs to improve their selectively and affinity to cancer cells. However, due to a broad range of possible choices of surface decorating molecules, it is difficult to choose the proper one for targeted functions. In this work, we will review several representative experimental and computational studies in selecting the proper surface functional groups. Experimental studies reveal that: (1) the NPs with surface decorated amphiphilic polymers can enter the cell interior through penetrating pathway; (2) the NPs with tunable stiffness and identical surface chemistry can be selectively accepted by the diseased cells according to their stiffness; and (3) the NPs grafted with pH-responsive polymers can be accepted or rejected by the cells due to the local pH environment. In addition, we show that computer simulations could be useful to understand the detailed physical mechanisms behind these phenomena and guide the design of next-generation NP-based drug carriers with high selectivity, affinity, and low toxicity. For example, the detailed free energy analysis and molecular dynamics simulation reveals that amphiphilic polymer-decorated NPs can penetrate into the cell membrane through the “snorkeling” mechanism, by maximizing the interaction energy between the hydrophobic ligands and lipid tails. We anticipate that this work will inspire future studies in the design of environment-responsive NPs for targeted drug delivery.

## 1. Introduction

Nanomaterials have been recognized as emerging materials in the design of drug delivery vehicles [1,2,3,4]. Due to the sequestration in the reticulo-endothelial system (RES) organs [5], degradation by serum protein absorption [6], macrophage internalization [7], and many other factors, the freely administrated drug molecules cannot be efficiently delivered to diseased cells. To overcome these biological barriers, nanomaterials have been found to be able to carry these drug molecules and effectively deliver them into tumor cells [8,9,10]. Langer and co-workers have developed a biodegradable long-circulating polymeric nanosphere based on amphiphilic copolymers [8]. When the polyethylene glycol-poly(lacticco-glycolic acid) (PEG–PLGA) copolymers are immersed in the water, their hydrophobic parts (PLGA) will self-assemble together to form a hydrophobic core region and minimize their interactions with water molecules. In the meantime, the hydrophobic drug molecules can also be enveloped into this core region with a 45% weight ratio. Due to the hydrophilic properties of PEG polymers, adsorption of serum proteins are blocked by these polymer brushes. Thus, the synthesized nanosphere has been found to exhibit long circulation time within blood flow and less accumulation in the liver of mice [8]. The above example demonstrates that nanomaterials have great potential in the design of a targeted drug delivery platform.

**Figure 1:**
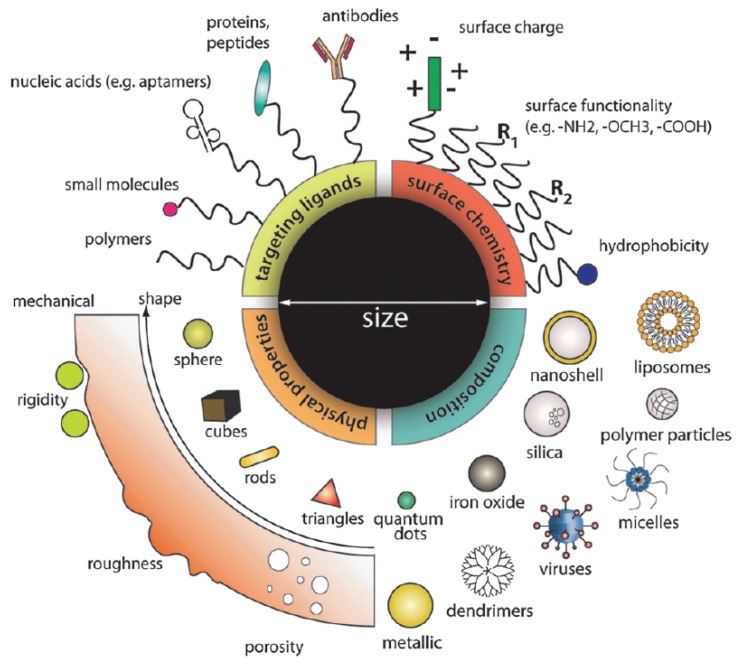
Design of nanoparticle (NP)-based drug delivery platform, according to the size, shape, stiffness (composition), and surface properties of NPs. The figure is adapted from Reference [11] with permission.

With the advancements in nanotechnology, size, shape, stiffness (composition), and surface properties of nanomaterials can be precisely controlled during synthesis. More importantly, these properties have been found to play important roles in the design of nanoparticle (NP)-based drug carriers with high efficacy [11,12,13,14,15,16,17,18], as summarized in Figure 1. Among them, surface functionalization has been considered as the most important factor. For instance, the bare inorganic NPs, such as Au or Ag NPs, can easily aggregate together in water, as they are hydrophobic. More importantly, when they are injected into the blood flow, the surfaces of inorganic NPs are usually attached with serum proteins due to electrostatic interactions. Thus, these particles will be visible to the immune cells (*i.e.*, macrophage) and eventually scavenged through phagocytosis. To overcome these issues, the biocompatible and hydrophilic PEG polymers have been widely used to decorate the surface of inorganic NPs. With a high grafting density, tethered PEG polymers form a brush on the surface of these NPs [19,20], and thus, they can be well-dispersed in the water. Also due to the grafted PEG chains, the absorption of serum proteins has been dramatically reduced [21,22]. Therefore, PEGylated NPs demonstrate prolonged circulation time and high accumulation in tumor sites *in vivo* due to the enhanced permeability and retention (EPR) effect [19,23,24,25].

The evolution in design of NPs for targeted drug delivery has been experienced in three generations [14,16]. The research about the first-generation NPs is focused on the basic surface chemistry, mainly the surface charges, to improve their biocompatibility and reduce toxicity. However, without considering the interactions between NPs and serum proteins, the first generation NPs can be removed quickly in the body by the immune cells. In comparison, the second generation of NPs are usually functionalized with bio-compatible polymers such as PEG. Under the protection of the tethered polymer layer, these particles could be able to exhibit a prolonged blood half-life time, which in turn helps these NPs to accumulate at the tumor site through the EPR effect [26,27]. In the recent development of the third-generation NPs, environment-responsive polymers have been adopted, by avoiding the over-reliance on the EPR effect. The local biological, physical or chemical cues are used to trigger the property change of tethered polymers and maximize the drug delivery efficacy. For example, the pH value in the tumor site (about 6) is relatively lower than that of normal tissue (about 7.4). Taking advantage of this acidic environment, the PEG surface shell could be removed by a pH-triggered effect to reveal the positively-charged inorganic NP core, facilitating the nonspecific cellular uptake of the drug-filled NPs [28]. Through the design of these “smart” polymers, the selectivity of NP-based drug carriers could be further enhanced.

The capability of NPs to survive during circulation is highly related to their surface properties. For example, neutral NPs have the longest blood circulation time compared with charged ones [14]. Based on the EPR effect, the NPs are able to extravasate through the loose vascular tissue to the tumor sites. Afterwards, the efficacy of the drug-loaded NPs is dictated by their internalization by the tumor cells, followed by the release of their payloads. As expected, cell membranes composed of amphiphilic lipids and membrane-associated proteins will be the main barrier to NP internalization. The pathway and efficiency of the internalization are highly related to the properties of the NPs, such as their size, shape, stiffness, and surface [29,30,31,32,33,34]. In short, to realize NP-mediated targeted drug delivery with high efficacy, it is necessary to carefully design the morphology and surface properties of NPs.

Compared to Au or silica NPs, magnetic nanoparticles (MNPs) like iron oxide NPs are attractive for biological or clinic applications due to their magnetic property [35]. For instance, they could be used for controlled drug delivery under external force [36,37], magnetic resonance imaging(MRI) [38], and magnetic thermotherapy [39]. However, on the other hand, the additional magnetic attraction between MNPs will lead to agglomeration. Further surface functionalization is the prerequisite for their stability in a biological environment [35]. As given in Table 1, the zwitterionic dopamine sulfonate polymer could be used to stabilize iron oxide NPs and reduce their affinity toward serum proteins [40]. The copolymers, such as CP-PEI, consisted of short chain polyethylenimine (PEI) and poly(ethylene glycol) (PEG) grafted to the natural polysaccharide chitosan (CP), and can be used to graft on the surface of iron oxide NPs [41]. The PEI–PEG–Chitosan-Copolymer-Coated iron oxide NPs are found to be able to efficiently load and protect nucleic acids and deliver the plasmid DNA in a C6 xenograft mouse model with high efficacy. The amphiphilic lipids or lipid-like molecules-coated MNPs will be biocompatible and have a great capacity for loading DNA or siRNA and delivering them through an external magnetic field [42].

From the above examples, we can see that the potential decorating molecules on the NP surface range from natural materials, such as nucleic acids, lipids, and proteins to synthetic polymers, like PEG, CP-PEI copolymer, 11-mercaptoundecane sulfonate (MUS), and octanethino (OT). These materials are the key parameters in the design of second- and third-generation NPs, which needs to be carefully evaluated. However, due to the limitation of current experimental techniques, many molecular mechanisms behind the design of these novel NPs are still not clear. Highlighting the important role played by the NP surface, we aim to review some representative researches in experiments and simulations on the design of NP surfaces. Experimental studies reveal that: (1) the NPs with surface-decorated amphiphilic polymers can enter the cell interior through penetrating pathway; (2) the NPs with tunable stiffness and identical surface chemistry can be selectively accepted by the diseased cells according to their stiffness; and (3) the NPs grafted with pH-responsive polymers can be accepted or rejected by the cells due to the local pH environment. In addition, we show that computer simulations could be useful to understand the detailed physical mechanisms behind these phenomena and may guide the design of next-generation NP-based drug carriers with high selectivity, affinity, and low toxicity. For example, the detailed free energy analysis and molecular dynamics simulation reveals that amphiphilic polymer-decorated NPs can penetrate into the cell membrane through the “snorkeling” mechanism, by maximizing the interaction energy between hydrophobic ligands and lipid tails. Since the cell membrane is one of the major barriers for NP-based drug carriers entering the interior of diseased cells, we will mostly focus on the molecular interaction between the surface-modified NPs and the cell membrane. We anticipate that this work will inspire future studies in the design of environment-responsive NPs for targeted drug delivery.

## 2. Experimental Studies

Although there are hundreds of different lipid molecules existing in nature, the majority of them are amphiphilic in the formed lipid bilayer of cell membrane, *i.e.*, the lipid heads are hydrophilic, while the lipid tails are hydrophobic. Besides, the cell membrane surface usually carries negative net charge due to its charged lipid heads. Considering these properties of the cell membrane, the hydrophobic, hydrophilic, and electrostatic properties of polymers and other organic molecules could be used to decorate the surface of NPs for their similarity in chemistry and physical interactions with the cell membrane.

### 2.1. Amphiphilic Polymer-Decorated NPs

**Figure 2:**
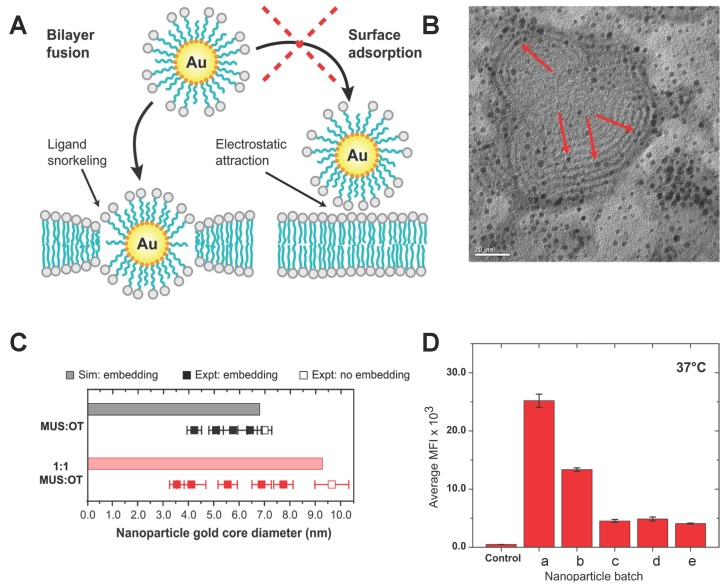
Penetration of the amphiphilic polymer decorated NPs and its dependence on NP diameter and surface composition. (**A**) Schematic description of the interaction between amphiphilic polymer-decorated NPs and the cell membrane; (**B**) Transmission electron microscopy (TEM) image of the giant multilayer vesicle interacting with 1:1 MUS:OT NPs. The red arrows point out that the Au NPs were inserted into the lipid bilayer. The average diameter of the NPs is 2.2 nm; (**C**) Filled-in squares represent experimental particles that successfully inserted, while empty squares specify those that did not; (**D**) HeLa cellular uptake in 37 ${}^{\circ }$C for all MUS NPs with different diameters, a (2.4±0.2 nm), b (2.9±0.5 nm), c (3.4±0.8 nm), d (4.9±1.1 nm), e (5.8±1.4 nm). The cellular uptake was measured by the median fluorescence intensity. The figures are adapted from References [52,54] with permission.

The Au NPs protected by an amphiphilic monolayer, with size below 10 nm in diameter, were reported to be able to enter the cell lin a non-disruptive way [44,52]. In the experiments [52], 11-mercaptoundecane sulfonate (MUS) with a negatively-charged end group, and octanethino (OT), were grafted on the Au NP’s surface. Both the MUS and OT are formed by an alkyl backbone with a sulfur head atom, while the other end of the MUS has a negatively-charged group. The 1:1 MUS:OT NPs interact with the giant multilayer vesicle (GMVs) and insert into the bilayer (see Figure 2A), keeping the vesicle intact. The existing inserting state in the inner bilayer of the GMVs suggested that the 1:1 MUS:OT NPs could penetrate and enter the vesicle, confirmed by transmission electron microscopy images (see Figure 2B). Here the GMVs was formed by zwitterionic 1,2-Dioleoyl-*sn*-glycero-3-phosphocholine (DOPC). The authors further explored the situations for vesicles with negative charge in the no-salt and salt solvent conditions. Similar behaviors were observed, indicating that the penetration of amphiphilic NPs was not induced by electrostatic interactions, as illustrated in Figure 2A. These results suggest that these amphiphilic polymer-decorated Au NPs can enter the vesicle or cell through direct penetration. Ignoring the electrostatic interactions, the hydrophobicity of alkyl backbones and the amphiphilicity of lipid molecules suggests that the penetration of amphiphilic NPs should be driven by the hydrophobic effect [53].

Through the hydrophobic effect, the interaction between amphiphilic NPs and the cell membrane should be affected by the decorated amphiphilic monolayer. Van Lehn *et al.* further studied the interaction between the “black” lipid membrane (BLM) and amphiphilic NPs with different monolayer compositions. They found that NPs with a certain range of diameters could be embedded into the cell membrane, as shown in Figure 2C. Besides, 1:1 MUS:OT NPs have a bigger cut-off threshold diameter than the 2:1 MUS:OT. It indicates that increasing the hydrophobic ligands (OT) might enhance the hydrophobic effect and facilitate the subsequent insertion. To further explore the NP size effect on penetration, the authors incubated HeLa cells with the all-MUS Au NPs with diameters ranging from 2.4 to 5.8 nm. As shown in Figure 2D, smaller amphiphilic NPs demonstrate higher cellular uptake efficacy. In addition, the number of NPs internalized by HeLa cells would not be affected beyond the diameter of 4.9 nm. All of the above observations suggest that the penetration of amphiphilic polymer-decorated NPs is highly related to the monolayer composition and size of NPs.

### 2.2. Lipid Molecule-Decorated NPs

**Figure 3:**
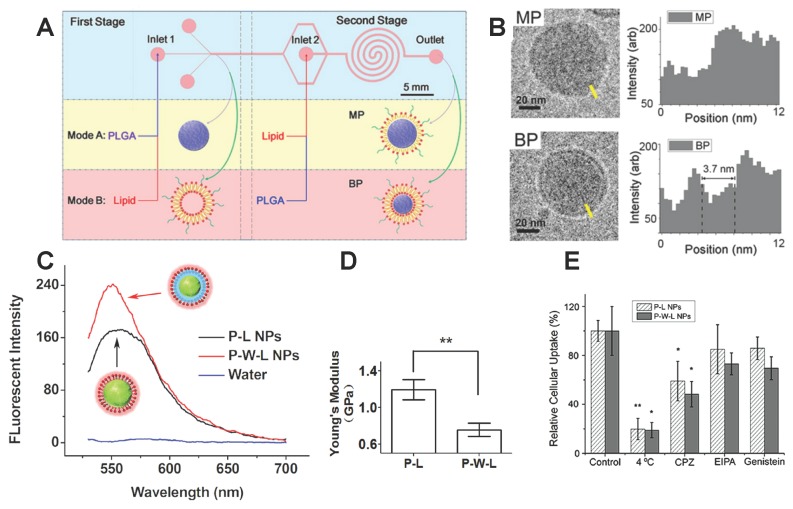
Structure difference in lipid molecule-decorated NPs and its effect on endocytosis. (**A**) Schematic of the two-stage-microfluidic platform; (**B**) Cryo-TEM images of monolayer shell lipid-polymer hybrid nanoparticles (MP P-L NPs) and bilayer shell lipid-polymer hybrid NPs (BP P-W-L NPs), the electron density showed that the BPs had a lipid bilayer. While the MPs have only one layer of lipids; (**C**) Plot of the fluorescence emission spectrum of BPs and MPs. It suggests that the BPs contain more water; (**D**) Plot of the Young’s moduli for MPs and BPs. MPs had higher Young’s modulus than the BPs with same size of 40 nm in diameter; **E**: The HeLa cells had a higher uptake of MPs than BPs. Chlorpromazine (CPZ), Ethylisopropyl amiloride (EIPA), and Genistein are used for inhibiting clathrin-mediated endocytosis, macropinocytosis, and caveolae-mediated endocytosis, respectively. The figures are adapted from References [50,51] with permission.

Comparing with MUS and OT ligands, lipid molecules are another natural choice for decorating the NP surface. It was reported that the lipid-polymer hybrid NPs (LNPs) would be another ideal drug carrier platform. Shi and co-workers [50,51] developed a two-stage microfluidic platform as shown in Figure 3A. Changing the injection order of poly(lactide-co-glycolide) (PLGA) and lipids, the PLGA would interact with the random lipids and liposome in modes A and B, respectively. These two modes could produce the LNPs covered by a monolayer-shell (MPs) or a lipid-bilayer shell (BPs). The structure of the bilayer and the monolayer were confirmed by the cryo-transmission electron microscope (Cryo-TEM), as depicted in Figure 3B. For MPs, the lipid molecules are in direct contact with the PLGA, while for BPs, there was a water layer between the inner PLGA core and the outer bilayer. The difference in water content between the MPs and BPs was further confirmed through the fluorescence emission spectrum (see Figure 3C). Due to the discrepancy in molecular structure, MPs and BPs can exhibit different mechanical properties. Through atomic force microscopy measurements, BPs demonstrate a smaller Young’s modulus than that of MPs of similar size, given in Figure 3D. The different mechanical properties of BPs and MPs might lead to different efficacy in cellular uptake, which is explored by incubating the HeLa cells with the MPs, BPs, and liposomes of similar size and surface chemistry. The results show that the number of internalized MPs was larger than that of BPs, presented in Figure 3E, indicating that the MPs could enter the HeLa cell more efficiently. These experiments also suggest that both MPs and BPs were internalized through clathrin-mediated endocytosis. The higher efficiency for cellular uptake of MPs are further confirmed in the *in vivo* experiments. The drug-loaded MPs can be more effective in controlling the growth of tumor cells.

### 2.3. pH-Responsive Polymer-Decorated NPs

**Figure 4:**
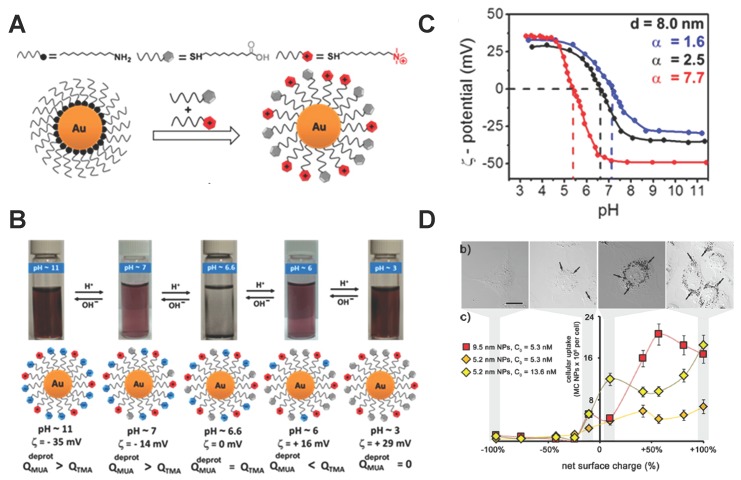
Properties of pH-responsive polymer-decorated NPs and their influence on cellular uptake efficiency. (**A**) Schematic of ligand exchange reaction between dodecylamine (DDA) NPs and mercaptoundecanoic acid (MUA), *N*,*N*,*N*-trimethyl(11-mercaptoun decyl)ammonium (TMA) ; (**B**) Images of solution under various pH values, containing 8.0 nm mixed-charged (MC) NPs with $\alpha_{\text{surf}}=2.5$. The MC NPs would be stable except at pH value 6.6. The scheme in the lower part illustrates the corresponding surface charge of the MC NPs, and it became zero net charge at pH value 6.6; (**C**) Plot of the changes of the *ζ* potential on the MC NP’s surface, relating to the solvent pH value and the ligand composition $\alpha_{\text{surf}}$. The diameter of the NP core was 8 nm; (**D**) Optical images of Rat 2 cellular uptake, indicated by the arrows. The cellular uptake increases with increasing net surface charge. The figures are adapted from Reference [55] with permission.

Apart from the hydrophobic and hydrophilic properties, the charge in the ligand might be another potential character used to design the surface properties of NPs. The NPs grafted with different ligands could carry various surface charges and demonstrate different cellular uptake efficiency [45]. Recently, Grzybowski and co-workers [55] reported that a kind of mixed-charged (MC) NP could adjust its surface charge according to the local pH value in the surrounding environment and exhibit distinct cellular uptake efficiency. The MC NPs, in their experiments, were synthesized through the ligand exchange reaction and were functionalized with positively charged *N*,*N*,*N*-trimethyl(11-mercaptoun decyl)ammonium ion (TMA) and neutral 11 mercaptoundecanoic acid (MUA), which have a carboxylic acid (COOH) group at the free end. Then, the carboxylic acid group could be deprotonated or protonated in the high or low pH value solvent, respectively, leading to negatively-charged and neutral properties (see Figure 4A).

Further experiments reveal that these MC NPs could be stable and their net surface charges are adjustable according to the solvent pH value pH${}_{\text{sol}}$. As shown in Figure 4B, the MC NPs demonstrate stability in a wide range of pH${}_{\text{sol}}$. Precipitation of MC NPs would only appear in a small region, when the surface net charge is zero. For a given MC NP, their net surface charge could decrease as the pH${}_{\text{sol}}$ increases, being positive and negative under low and high pH${}_{\text{sol}}$ values, respectively. Furthermore, for a certain size of MC NP, its sensitivity to the pH${}_{\text{sol}}$ depends on the composition ratio of MUA and TMA $\alpha_{\text{surf}}=c_{\text{surf}}^{\text{MUA}}/c_{\text{surf}}^{\text{TMA}}$ (see Figure 4B), indicating that the desired surface net charge at a given pH${}_{\text{sol}}$ can be obtained by altering $\alpha_{\text{surf}}$. Moreover, incubating the Rat 2 fibroblasts with various-sized MC NPs demonstrates that the internalization of these NPs is highly dependent on their size and surface net charge. As shown in Figure 4D, the positively charged MC NPs are favorable for cellular uptake, while the negative ones cannot be internalized. For the given surface net charge, increasing the NP size will facilitate cellular uptake. Considering the physiological condition that the pH value in the tumor extra-cellular environment is lower (about 5–6) than the normal pH value (about 7.4) in the human body, these MC NPs with stability in different pH${}_{\text{sol}}$ solvent and adjustable surface net charge could be used as ideal “smart” drug carriers for targeting cancer cells.

## 3. Computational Studies

From the above representative experiments, it is clear that the surface properties of NPs can be modified to facilitate their selective interaction with normal and diseased cells, which opens the opportunity for targeted drug delivery. However, due to the limitation of the current experimental technology, more details, such as the penetration and internalization process of NPs, cannot be directly observed and quantified through experiments. To resolve this issue, computer simulations provide a useful tool to approach these details and uncover the fundamental physical mechanisms. Taking advantage of these simulations, the important role played by the surface-decorated molecules on NPs could be identified. Understanding these important mechanisms will also provide us with guidelines in the design of novel NP-based drug carriers with high selectivity and affinity to tumor cells. In the following part, we will demonstrate three examples in understanding the surface properties of NPs and their interactions with cell membranes through computer simulations.

### 3.1. Amphiphilic Polymer-Decorated NPs

**Figure 5:**
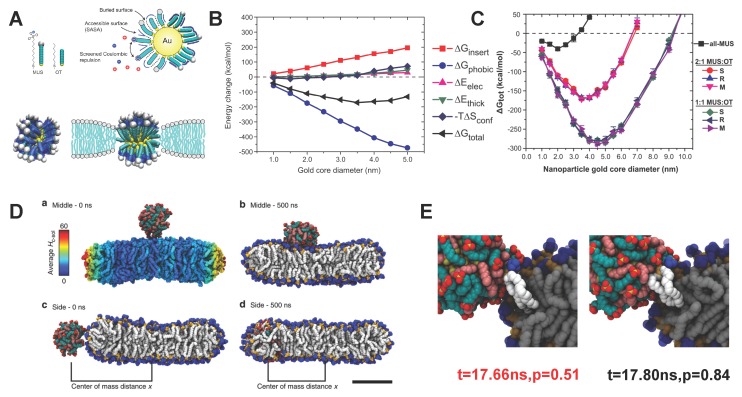
Free energy and kinetic barrier analysis for penetration of amphiphilic polymer-decorated NPs. (**A**) (**Upper** part) Schematic of the solvent-accessible surface area (SASA), a parameter of the hydrophobic free energy, and electrostatic interactions; (**Lower** part) comparison between the initial and final stable states of the amphiphilic polymer-decorated NPs. The hydrophilic ligands are squeezed out of the bilayer’s hydrophobic region, forming a so-called “snorkeling” phenomenon; (**B**) Decomposition of the free energy during the NP penetration process. $\Delta G_{\text{total}}$ is the total free energy change. $\Delta G_{\text{phobic}}$ is the change of hydrophobic energy, determined through the SASA. $\Delta E_{\text{elec}}$ is the electrostatic energy change. $\Delta E_{\text{thick}}$ is the free energy change corresponding to deformation of the lipid bilayer. $\Delta S_{\text{conf}}$ is the conformation entropy change of the decorated amphiphilic polymers; (**C**) Total free energy change $\Delta G_{\text{total}}$ as a function of NP diameter and monolayer composition. The ligand density on the Au NPs is kept constant; (**D**) Snapshots of the initial and final states of the NPs interacting with a lipid bilayer. For the case of 1:1 MUS:OT NP on the top of the bilayer, it cannot penetrate into the bilayer (**Upper** part); while it can penetrate in the bilayer through the edge (**Lower** part); (**E**) Snapshots of the transition states during NP penetration through the edge of a bilayer. At the transition time (*t* = 17.66 ns), the protruding lipids were in contact with the amphiphilic NP. The figures are adapted from References [52,54,57,63] with permission.

According to the experiments by Van Lehn *et al.* [52], the penetration of amphiphilic polymer decorated NPs into a lipid bilayer is driven by the hydrophobic effect. The insertion state of amphiphilic NPs could bring other changes into the solvent-membrane-amphiphilic NPs (SMAN) system. Due to the low dielectric constant within the bilayer’s hydrophobic core region, direct contact is energetically unfavorable for the hydrophilic ligands with positive charges. Therefore, they are assumed to change their configurations. Moreover, the mismatch of properties between amphiphilic NPs and the bilayer’s hydrophobic core region could also introduce deformation into the lipid bilayer. The electrostatic interactions will also be changed due to the configuration change of the ligands and membrane. Through the Monte Carlo (MC) simulation with implicit water model, Alexander-Katz with co-workers analyzed the detailed free energy change of each component between the initial and final states of the SMAN system. As the schematic configurations are shown in Figure 5A, it is clear that the hydrophilic ligands on the surface of a Au NP were squeezed out, keeping the anionic head groups away from the hydrophobic region of the bilayer. Such a phenomenon is referred to as “snorkeling”, being anomalous to the characteristic of protein insertion [56]. It should be emphasized that the “snorkeling” mechanism is highly related to the flexibility of ligands [57]. As presented in Figure 5B, the free energy change $\Delta G_{\text{phobic}}$ induced by the solvent-accessible surface area (SASA) provides the driving force during this process. Thus, the total energy change of the system $\Delta G_{\text{total}}$ will be reduced during the penetration process, facilitated by the reduction of $\Delta G_{\text{phobic}}$. Besides, the composition of MUS and OT ligands also plays an important role, as shown in Figure 5C. With the same grafting density of ligands and NP diameter, $\Delta G_{\text{total}}$ for the NPs with 1:1 MUS:OT is much lower than that of the 2:1 MUS:OT and all MUS NPs. It provides an excellent explanation as to why 1:1 MUS:OT NPs can more easily insert into the lipid bilayer than other NPs, as being observed in the previous experiments (see Figure 2C). More importantly, the total free energy change $\Delta G_{\text{total}}$ can become positive if the diameter of the core reaches a certain value, indicating that the insertion of amphiphilic NPs is energetically unfavorable. These values of the diameters are denoted as the cut-off threshold. Furthermore, decreasing the composition of hydrophobic ligands (MUS) will increase this threshold, as given in Figure 2C. All these simulation results are in good agreement with previous experimental observations (see Figure 2), providing in-depth mechanisms into these phenomena. Apart from the NP size and surface composition, many other factors, including the ligand length, ligand rigidity, grafting density, and distribution morphology of ligands could also affect the total free energy change $\Delta G_{\text{total}}$ during the penetration process [52,54,57,58,59,60,61], and eventually change the cut-off threshold for the amphiphilic NPs penetration. The above MC simulation can only reveal the thermodynamic features before and after penetration. To understand the dynamics during this process, Van Lehn *et al.* have performed atomistic molecular dynamics (MD) simulations [59]. In these MD simulations, when the initial position of the amphiphilic NPs are close to the ribbon bilayer edge, they can successfully insert into the bilayer. While the penetration has been prohibited, when the amphiphilic NPs were in the middle bilayer planar as the initial configuration, given in Figure 5D. The committor analysis has been used to analyze the transition state for understanding this difference. As illustrated in Figure 5E, a protruding lipid in the bilayer ribbon edge interacted with the hydrophobic part of the ligands and remained in contact during the whole process of penetration. Thus, it indicated that the lipid protruding might be a necessary condition for amphiphilic NPs penetrating into the bilayer. The lipid protrusion itself has an energy barrier as 10.5 *k*${}_{B}$*T*, which is relatively high for the normal fluctuation near the bilayer middle plane. Here *k*${}_{B}$ and *T* are the Boltzmann constant and temperature, respectively. Therefore, it could be a main energy barrier during the kinetic process. The necessity of the lipid protrusion for amphiphilic NPs penetration was further confirmed in their later work [58] and a following study [62].

### 3.2. Lipid Molecule-Decorated NPs

**Figure 6:**
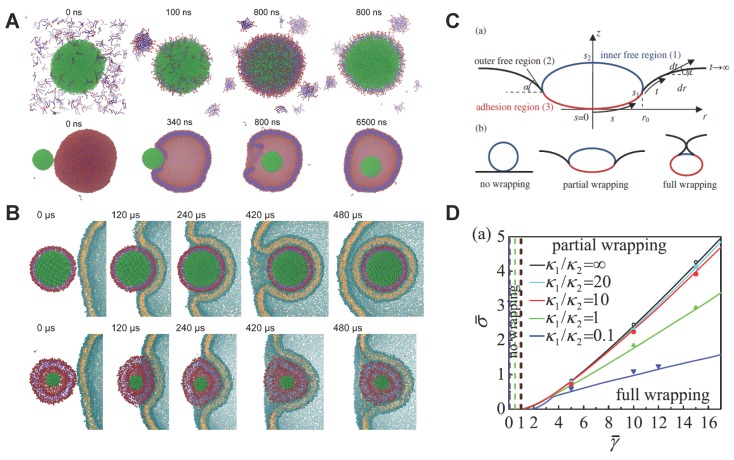
Self-assembly and subsequent endocytosis of lipid molecule-decorated NPs (MPs and BPs). (**A**) Computational modeling on the self-assembly process of the modes A and B given in Figure 3A. In mode A (**Upper** part), the NP core could interact with the random lipids and form the MPs. While in mode B (**Lower** Part), the NP core will interact with the pre-assembled liposome and form the BPs; (**B**) Snapshots of the MPs and BPs interacting with a vesicle. Upper and lower parts are corresponding to the MPs and BPs, respectively; (**C**) Schematic of different states of the endocytosis of soft NP; (**D**) Phase diagram for the endocytosis of NPs with different stiffness. $\kappa_{1}$ and $\kappa_{2}$ are the bending rigidities of the NP and membrane, respectively. The solid lines represent the phase boundaries between the fully wrapping and partial wrapping regimes. The horizontal axis represents the adhesion energy $\bar{\gamma }$ and the vertical one denotes the membrane tension $\bar{\sigma }$. These figures are adapted from References [51,64] with permission.

As aforementioned, both the MPs and BPs can be synthesized through the microfluidic system. MPs present higher rigidity than BPs, and demonstrate higher cellular uptake. To understand their different mechanical behaviors and related biological response, Shi and co-workers have performed coarse-grained MD simulations on these systems to understand their self-assembly process and endocytosis [50,51]. As shown in Figure 6A, when PLGA NPs are mixed with random lipid molecules in water, the lipids can form a monolayer on the surface of the PLGA core, due to the hydrophobic properties of PLGA and lipid tails. In contrast, when the PLGA NPs are mixed with the pre-assembled liposome, the lipid tails were not visible to PLGA NPs. The PLGA NP can gradually contact and enter the interior of the liposome, forming the BPs (see Figure 6A). By fixing the size and surface chemistry of MPs and BPs, the authors further explored their endocytosis. The simulation results reveal that the MPs can easily enter into the cell, while the BPs were trapped on the surface of membrane, as shown in Figure 6B. By carefully investigating the simulation results, the authors find that BPs with lower stiffness could spread on the surface of the membrane (at 120 μs in Figure 6B) with significant deformation. However, MPs with higher stiffness did not deform as much as BPs and are mostly accompanied by membrane deformation. During the late stage (420 μs), the soft NPs (BPs) encounter larger deformation of the membrane with high energy barriers. Therefore, it is more difficult for BPs to enter the interior of the cell, which are consistent with the previous experimental observation (see Figure 3E).

The distinction in the deformation denotes that BPs would encounter a larger energy barrier for the fully wrapping. These results could be explained by the theory model developed by Yi *et al.* [64]. As in the schematic shown in Figure 6C, the endocytosis of an elastic NP could be simplified as a deformable vesicle with a constant surface area in contact with an initially flat membrane. Three different stages could be considered during the internalization: no wrapping, partial wrapping, and full wrapping. Considering the elastic deformation energies of the NP and cell membrane, as well as the adhesion energy between the NP and membrane, the total energy of the system could be theoretically formulated. By minimizing this total energy with proper boundary conditions, the equilibrium configurations of the vesicle and membrane can be numerically solved. According to the different values of the membrane tension $\bar{\sigma }$, adhesion energy $\bar{\gamma }$, vesicle bending rigidity $\kappa_{1}$, and membrane bending rigidity $\kappa_{2}$, a phase diagram can be theoretically obtained to characterize the boundaries between the no wrapping, partial wrapping, and full wrapping states, as shown in Figure 6D. From this phase diagram, it is clear that under the given membrane tension $\bar{\sigma }$, the soft NPs (smaller value of $\kappa_{1}$) requires la arger value of the adhesion energy $\bar{\gamma }$, compared to stiff NPs (larger value of $\kappa_{1}$). Therefore, the internalization of soft NPs will be more difficult than stiff NPs under the same physiological condition, as the soft NPs tend to spread on the membrane surface and encounter larger energy barriers.

### 3.3. PEGylated NPs

**Figure 7:**
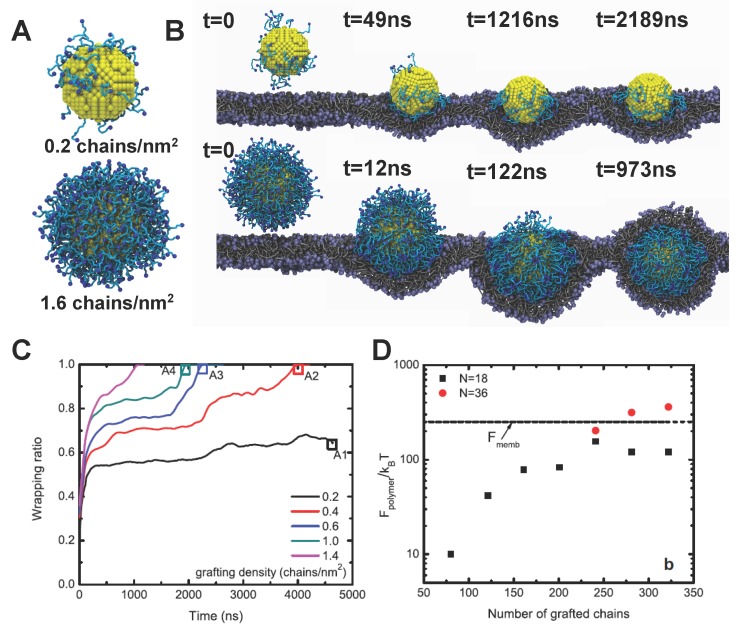
Internalization process of PEGylated NPs and related free energy change. (**A**) Snapshots of PEGylated NPs used in the dissipative particle dynamics (DPD) simulations with two grafting densities: 0.2 and 1.6 chains/nm${}^{2}$. The diameter of the NP core is about 8 nm. The polymerization degree of tethered chains is 18, corresponding to the molecular weight 838 Da; (**B**) Endocytosis of PEGylated NPs with different grafting densities. When the grafting density is low, *i.e.*, 0.2 chains/nm${}^{2}$, the NP will be trapped on the surface of membrane. While for high grafting density, 1.6 chains/nm${}^{2}$, the NP will be fully wrapped by the membrane; (**C**) Wrapping ratio versus time for PEGylated NPs with different grafting densities; (**D**) Free energy change of the tethered polymers during endocytosis. The dashed line represents the bending energy of the membrane.

To understand the internalization of PEGylated NPs, Li *et al.* have performed large scale dissipative particle dynamics (DPD) simulations [65,66]. Through a systematic coarse-graining process [67,68], an accurate DPD model has been developed for PEG polymers, which can reproduce their end-to-end distance $R_{\text{ee}}$ and radius of gyration $R_{g}$ in water. Thus, the conformation of PEG chains and PEGylated NPs can be correctly represented in the DPD simulations, depicted in Figure 7A. The core of the NP was considered to move as a rigid body during the DPD simulation. All the relevant intermolecular interactions between different molecules have also been calibrated through experiments [65]. To mimic the experimental condition, all the free ends of tethered chains are covalently bound with targeting moieties, which can specifically recognize and bind with receptors expressed on the surface of the cell membrane.

The typical internalization process of PEGylated NPs with different grafting densities is given in Figure 7B. When the grafting density is low, such as 0.2 chains/nm${}^{2}$, the PEGylated NP will mainly be absorbed on the surface of membrane without membrane wrapping. However, when the grafting density is high enough, such as 1.6 chains/nm${}^{2}$, the PEGylated NP will be firstly wrapped around by the lipid bilayer, followed by the membrane extrusion, and eventually fully wrapped by the lipid bilayer. These distinct behaviors indicate that the grafting density of PEG polymers could play important roles during the endocytosis. Besides, the wrapping ratio of PEGylated NPs with different grafting densities are plotted against time in Figure 7C. Clearly, the critical time for the PEGylated NPs to be fully wrapping is highly dependent on the grafting density. With the grafting density of PEG decreasing, the wrapping time could be dramatically enlarged, finally leading to the non-wrapping state.

To understand these different behaviors, the free energy analysis has been carried out by Li *et al.* [65,66]. Three major contributions have been identified for the free energy change of the system [12,65]. The first one is the change of the specific ligand–receptor interaction $\Delta F_{\text{ligand}}$, which provides the driving force for the PEGylated NPs to be internalized. Since all the free ends of tethered chains are attached with targeting moieties, $\Delta F_{\text{ligand}}$ is proportional to the grafting density (total number) of PEG polymers. The second is the membrane bending energy change $\Delta F_{\text{memb}}$, which is determined through the curvature of the membrane, and is equal to 8πκ if the spherical NP is fully wrapped. The last one is the non-specific free energy change of the tethered PEG polymers $\Delta F_{\text{polymer}}$, which is induced by the configurational entropy change of PEG during endocytosis. With the help of self-consistent field (SCF) theory [66,69,70], $\Delta F_{\text{polymer}}$ is found to be composed of elastic ($\Delta F_{\text{el}}$) and interaction ($\Delta F_{\text{int}}$) parts. The elastic part ($\Delta F_{\text{el}}$) originates from the compression or tension of the PEG chains, considering the polymer chain as an entropic spring. Therefore, $\Delta F_{\text{el}}$ is directly reflected through the mean squared end-to-end distance, $R_{ee}$, of tethered chains. $\Delta F_{\text{int}}$ arises from the intermolecular interaction between different chains, characterized through the radial volume fraction profile ϕ(r). Therefore, when the driving force $\Delta F_{\text{ligand}}$ is larger than the sum of $\Delta F_{\text{memb}}$ and $\Delta F_{\text{polymer}}$, fully wrapping of PEGylated NPs will be energetically favorable.

As given in Figure 7D, $\Delta F_{\text{polymer}}$ increases with increasing grafting density. More importantly, $\Delta F_{\text{polymer}}$ could be comparable with the membrane bending energy $\Delta F_{\text{memb}}$, when the grafting density is high enough. When the tethered chains are long enough, $\Delta F_{\text{polymer}}$ could be larger than $\Delta F_{\text{memb}}$. This important finding signals that wrapping of PEGylated NPs could be greatly affected by the entropy change of tethered chains. Considering the large molecular weight of PEG used in experiments, the $\Delta F_{\text{polymer}}$ could dominate the endocytosis of PEGylated NPs. In addition, to clarify the shape effect during internalization of PEGylated NPs, spherical, rod-like, cubic, and disk-like NPs have been computationally studied [66]. Under the equal surface area of NP core and grafting density of PEG polymers, the spherical NPs demonstrate the fastest internalization rate, followed by the cubic NPs, rod-like NPs, and finally disk-like NPs, due to the different membrane bending energies encountered [66]. It is worth noting that the aforementioned simulation is mainly based on the interaction between a model bilayer membrane and PEGylated NPs. However, the endocytosis of NPs may involve other mechanisms (e.g., coveolae-mediated, clatherin-mediated endocytosis, micropinocytosis, *etc.* ) more complicated than membrane fusion. *In vitro* experiments have shown various results that higher cellular uptake due to anisotropy of PEGylated nanoparticles (silica [72], lipid mixtures [73], polymer nanoparticles [74,75,76]). For more detailed information, readers can refer to a good review article in [77]. To date, the fundamental understanding of shape-dependent uptake remains unclear.

## 4. Conclusion and Perspective

In this work, we have reviewed the recent progress in the design of NP surface properties to achieve targeted drug delivery with high efficacy. The experimental results reveal that the hydrophobic, hydrophilic, and charge properties of organic molecules could be utilized, mimicking the properties of lipids. For example, direct penetration without membrane disruption could be realized through amphiphilic polymer decoration. The lipid molecules come in contact with the hydrophobic polymer (PLGA) to form monolayer or bilayer lipid-coated NPs with different stiffness. The monolayer lipid coated NP (MPs) are found to be more efficiently taken up by the HeLa cells. The mixed charged (MC) NPs can display different surface charges according to the local pH environment, due to the protonation and deprotonation of the carboxylic acid group. Thus, the MC NPs can be more easily taken up when they display positive net surface charges. In addition, computer simulations provide useful tools to understand the fundamental mechanisms and reveal the molecular details about the penetration/endocytosis of NPs. With the help of these simulations, it was found that the ligand composition, flexibility of decorated molecules, and grafted polymer chain length and density are important factors governing the internalization of NPs. The hydrophobic energy and surface electrostatic interaction is determined by the composition of ligands (MUS and OT). With the hydrophobicity of the NP surface increasing (large ratio of MUS over OT), the amphiphilic NPs could more easily penetrate into the cell membrane. The flexibility of decorated molecules on the NP surface determines the membrane bending energy and elastic energy of NPs during the endocytosis of lipid coated NPs. Besides, the free energy change of tethered PEG chains is found to be comparable or even larger than that of membrane bending energy, which could greatly reduce the cellular uptake efficacy of PEGylated NPs. In-depth understanding of these mechanisms yields guidelines for researchers in designing the surface properties of NPs with high selectivity and affinity to diseased cells.

**Figure 8:**
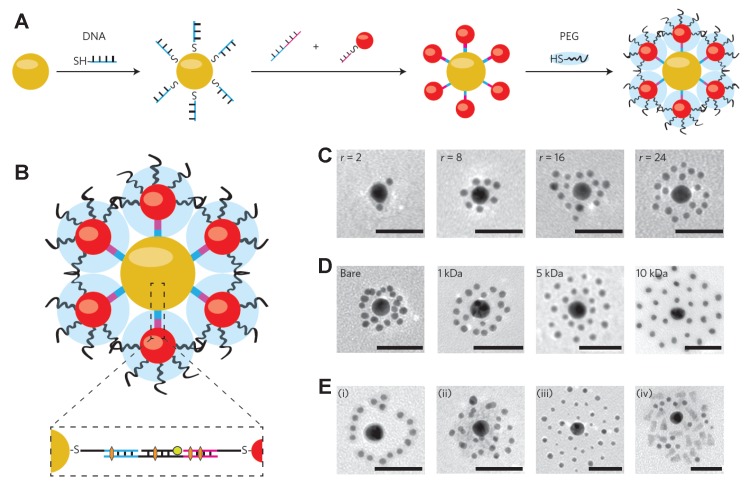
Design of NP superstructure by using the DNA linkers for self-assembly. (**A**) Individual NPs (**red** and **yellow** spheres) were coated with thiolated and single-stranded DNA and then self-assembled together due to the complementary DNA sequence. The surface of the assembled NP can be further decorated by PEG polymers (blue clouds) to control interactions with cells and tissues; (**B**) Cross-sectional view of the self-assembled NP superstructure of a core-satellite. The insert shows that the payloads could be encapsulated either via hybridizing (**green** circle) or intercalating (**orange** hexagon) to the DNA linkers; (**C**) TEM images of the NP superstructure of two-layer core satellites as a function of the satellite-to-core ratio (*r* = 2, 8, 16, and 24); (**D**) TEM images on the NP superstructure of two-layer core satellites as a function of the satellite PEG length (molecular weight $M_{w}$ = bare, 1, 5, and 10 kDa); (**E**) TEM images of the NP superstructure of three-layer core satellites by introducing the third DNA sequence. The scale bars for TEM images are 50 nm. The figure is adapted from Reference [83] with permission.

To realize NP-mediated drug delivery with high efficacy, decorating the NP surfaces with single molecules or polymers with simple structure might not be enough. For example, it is well-known that the NPs with large size (100–200 nm in diameter) could be more easily accumulated at the tumor sites through the EPR effect [18,78,79], while it will be easier for NPs with smaller size (20–50 nm in diameter) to be quickly accepted by diseased cells [48,80,81]. More importantly, the smaller-sized NPs with diameters below 10 nm can be cleaned and eliminated through a fenestrated endothelium in the spleen and kidney [82]. Considering these different length scale requirements, a single design principle will not work. To overcome this issue, Chou *et al.* proposed the use of different-sized NPs with surface coated DNA linkers to self-assemble together, forming a NP superstructure of a core-satellite, as shown in Figure 8A. The surface of this NP superstructure can be further modified by PEG polymers to control its interaction with cells and tissues. The payloads can be encapsulated into the DNA linker either through hybridizing or intercalating, depicted in Figure 8B. The self-assembled NP superstructures could be easily controlled through the satellite-to-core ratio, satellite PEG length, and additional DNA sequence, evidenced by the TEM images shown in Figure 8C–E. Due to the large size of the overall NP superstructure and surface-decorated PEG polymers, the macrophage uptake has been reduced, accompanied by the tumor accumulation *in vivo* through the EPR effect [82]. More importantly, the NP superstructure degrades into building blocks when it approaches the tumor cells, releasing the payloads, and subsequently escape biological sequestration due to their smaller size. Other examples include a multilayer of polymers tethered on the surface of NP with different properties, such as pH-sensitive and tumor targeting [28]. The design of these novel NPs involves multiple design principles, based on the key features of the NP surface functionalization, revealed through the experimental and computational studies. By discussing how the basic physical and chemical properties of organic molecules could be used to design the NP surface, and what could be the governing parameters/factors during the internalization process, we expect that this work could inspire further studies in the design of the environmental-responsive NPs for drug delivery with high efficacy.

## Figures and Tables

**Table 1:** Examples of various nanoparticle surface modifications.

Category	Surface	Core	Key Observation	References
Polymer	PEG *	Au *, liposome	Long blood-circulation time, different celluar uptake pathway	[19,23,43]
Polymer	MUS *, OT *	Au	Direct penetration without membrane disruption	[44]
Polymer	AP *, GP *, GEGP *, FAP *	MS *	Surface charge related cellular uptake efficiency	[45]
Polymer	ZDS *	SPIO	High stability, reduced nonspecific affinity	[40]
Copolymer	CP-PEI *	SPIO *	High efficiency in DNA delivery	[41]
DNA	Oligonucleotide	Au	High efficiency in cellular uptake mediated by absorbed protein	[46]
Protein	Transferrin	Au	Clathrin-mediated endocytosis pathway and non-toxicity	[47,48,49]
Lipid	Various lipids	SPIO	High stability and drug capacity, good cellular uptake	[42]
Lipid	DPPC *	PLGA *	Cellular uptake depends on the core–shell structure	[50,51]

* Abbreviation: PEG, poly-(ethylene glycol); Au, gold; MUS, 11-mercaptoundecane sulfonate; OT, octanethino; AP, 3-aminopropyl; GP, guanidinopropyl; MS, mesoporous silica; GEGP, 3-[*N*-(2- guanidinoethyl)guanidino]propyl; FAP, h-folate-3-aminopropyl; CP-PEI, chitosan-polyethylenimine; SPIO, superparamagnetic iron oxide; ZDS, zwitterionic dopamine sulfonate; PLGA, poly(lactic-*co*-glycolide); DPPC, 1,2-dipalmitoyl- *sn*-glycero-3-phosphocholine.
